# Pain clinic definitions in the medical literature and U.S. state laws: an integrative systematic review and comparison

**DOI:** 10.1186/s13011-018-0153-6

**Published:** 2018-05-22

**Authors:** Barbara Andraka-Christou, Joshua B. Rager, Brittany Brown-Podgorski, Ross D. Silverman, Dennis P. Watson

**Affiliations:** 1 Department of Health Management and Informatics, College of Health and Public Affairs, University of Central Florida, 4364 Scorpius Street, Orlando, FL 32816 USA; 2 School of Medicine, Indiana University, 340 W 10th St #6200, Indianapolis, IN 46202 USA; 3 Department of Social and Behavioral Sciences, Indiana University Fairbanks School of Public Health, 1050 Wishard Blvd, Indianapolis, IN 46202 USA; 4 Department of Health Policy and Management, Richard M. Fairbanks School of Public Health, Indiana University, 1050 Wishard Blvd, Indianapolis, IN 46202 USA; 5 Department of Social and Behavioral Science, Richard M. Fairbanks School of Public Health, Indiana University, 1050 Wishard Blvd, Indianapolis, IN 46202 USA

**Keywords:** Pain clinic, Pain treatment, opioid, state policy, Integrative review, Systematic review, Content analysis

## Abstract

**Background:**

In response to widespread opioid misuse, ten U.S. states have implemented regulations for facilities that primarily manage and treat chronic pain, called “pain clinics.” Whether a clinic falls into a state’s pain clinic definition determines the extent to which it is subject to oversight. It is unclear whether state pain clinic definitions model those found in the medical literature, and potential differences lead to discrepancies between scientific and professionally guided advice found in the medical literature and actual pain clinic practice. Identifying discrepancies could assist states to design laws that are more compatible with best practices suggested in the medical literature.

**Methods:**

We conducted an integrative systematic review to create a taxonomy of pain clinic definitions using academic medical literature. We then identified existing U.S. state pain clinic statutes and regulations and compared the developed taxonomy using a content analysis approach to understand the extent to which medical literature definitions are reflected in state policy.

**Results:**

In the medical literature, we identified eight categories of pain clinic definitions: 1) patient case mix; 2) single-modality treatment; 3) multidisciplinary treatment; 4) interdisciplinary treatment; 5) provider supervision; 6) provider composition; 7) marketing; and 8) outcome. We identified ten states with pain clinic laws. State laws primarily include the following definitional categories: patient case mix; single-modality treatment, and marketing. Some definitional categories commonly found in the medical literature, such as multidisciplinary treatment and interdisciplinary treatment, rarely appear in state law definitions.

**Conclusions:**

This is the first study to our knowledge to develop a taxonomy of pain clinic definitions and to identify differences between pain clinic definitions in U.S. state law and medical literature. Future work should explore the impact of different legal pain clinic definitions on provider decision-making and state-level health outcomes.

## Background

An estimated 25 million people suffer from chronic non-cancer pain in the United States [1]. Prescription opioids are commonly prescribed to treat chronic non-cancer pain but opioid therapy is associated with risks of abuse, misuse, and overdose. In 2013, an estimated 2 million Americans misused or abused prescription opioids [2]. Nonmedical use of prescription opioids is associated with high rates of emergency department visits, treatment admissions, and fatal overdoses [3]. From 1999 to 2013, prescriptions for opioid painkillers quadrupled, as did opioid-related deaths in the United States [4]. Despite the recent increase in heroin-related deaths, prescription opioids (including fentanyl) are involved in more opioid overdoses than any other drug [5]. A majority of long-term opioid misusers report obtaining their drugs from a doctor or from friends or relatives [6]. There is also growing evidence of a relationship between non-medical use of prescription opioids and heroin abuse, as individuals switch from prescription opioids to heroin [2, 3].

Many states have adopted a range of legislative and regulatory provisions with the goal of improving monitoring and oversight of opioid prescribing and dispensing [7]. These provisions seek to define the scope of practice for prescribers and dispensers, mandate dosage thresholds for opioid medications, and establish non-opioid treatment guidelines. Additionally, 49 of 50 states have established prescription drug monitoring programs (PDMPs) to facilitate provider prescribing of controlled substances, reduce overprescribing of opioid analgesics, and prevent illicit drug use and abuse [8]. Further, some state legislatures have enacted legislation and regulations specifically targeting health care facilities that primarily manage and treat chronic pain, namely pain clinics, as opioids are commonly prescribed in such clinics, sometimes without adequate justification or medical evaluation [9].

Whether or not a pain management facility is captured by a state’s pain clinic definition affects the extent to which it will be subject to state oversight and regulation. Furthermore, state pain clinic definitions may reveal policy priorities by encouraging some treatment modalities over others. One might expect state pain clinic definitions to be guided by the scientific medical literature; however, it is unclear whether they are. Prior research suggests discrepancies exist between the academic medical literature and what appears in state policy [10, 11]. Identifying such discrepancies in this context could assist states in designing pain clinic laws that are more compatible with scientifically informed best practices.

We conducted an integrative systematic review to understand the extent to which pain clinic definitions in the medical literature were reflected in existing state statutes and regulations. Our guiding questions include the following: How does the medical literature define pain clinics? To what extent are medical definitions reflected in state laws? This research is the first step in a larger project to evaluate state-level opioid policies in Indiana for which an operational definition of pain clinics was needed.

## Methods

We first describe the integrative review process and then how we identified existing state policies and applied the taxonomy to them. All searches and analyses related to this work were performed in MAXQDA qualitative analysis software [12].

### Integrative review of the medical literature

Integrative reviews are particularly useful for generating theory and developing constructs for facilitating understanding of complex problems [13–15]. An integrative review consists of: 1) problem formulation, 2) data collection, 3) evaluation of data, 4) data analysis, and 5) interpretation and presentation of the results [15]. Each of the first four steps of this process we carried out is detailed below, and the interpretation and presentation of the data is included in the results.

#### Medical literature data collection

Using Pubmed, Google Scholar, PsychINFO, and SCOPUS, we applied more than 10 Boolean search queries (see Table 1) to identify all scholarly literature pertaining to pain clinics. We limited our queries to academic literature and reports, evaluations, and white papers from medical organizations and advocacy groups (i.e., grey literature). A cursory scan of the academic literature revealed two seminal works on this topic [16, 17]; therefore, we employed a time parameter to limit the search to literature published between 1990 (when the seminal works were published) and 2016.^1^ Additionally, we only included articles written in English and that focused solely on pain management in humans. Lastly, due to our interest in conflicts between national standards and state laws, our grey literature search only included materials from U.S. medical organizations that specialize in pain management.

**Table 1:** Databases and search terms

Database	Search terms	Filters applied
PubMed (Medline)	“Pain Clinics/classification”[Majr] OR “Pain Clinics/standards”[Majr]) OR (“Pain Clinics/statistics and numerical data”[Majr] OR “Pain Clinics/trends”[Majr])	“Humans” and “Full-text” filters applied
Google Scholar	“pain clinics” OR “pain treatment” facility	
PsychINFO	(“pain treatment” OR “pain clinic”) AND facility	“Humans” and “English” filters applied.
SCOPUS	Citation chains for: (1) Loeser, et al. [16] (2) Wells & Miles [61]	

##### Academic literature

**Fig. 1:**
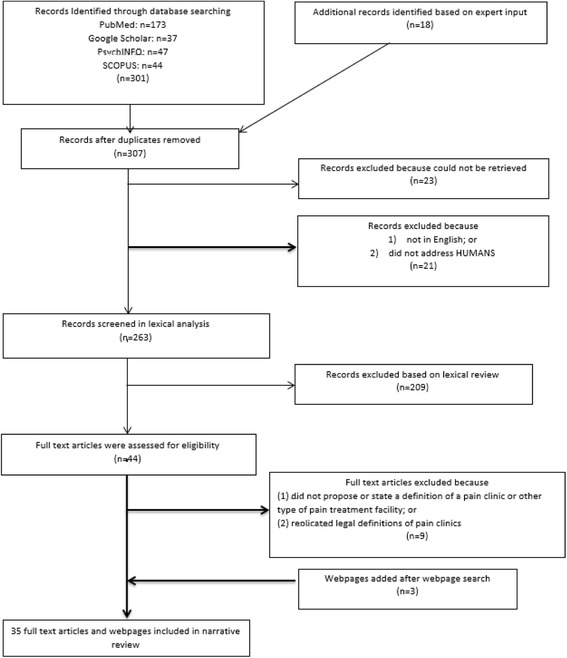
Medical literature search process

**Fig. 2:**
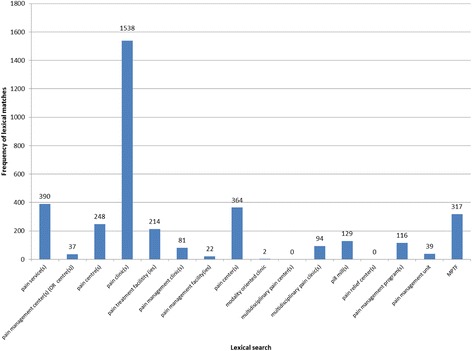
Frequency of lexical matches by term searched

Our initial search and inclusion criteria yielded 298 academic records, which we processed using EndNote bibliographic management software [18]. We identified and removed 12 duplicates from our initial sample. An additional 23 records were excluded because a full-text PDF could not be located.^2^ Our remaining sample (*n* = 263 articles) was loaded into MAXQDA for analysis (see Fig. 1) [12]. We conducted a targeted lexical search for specific key terms contained within each full-text record to determine eligibility for inclusion in this review. We utilized the following key terms: (1) pain clinic; (2) pain treatment facility OR pain treatment facilities; (3) pain management clinic; (4) pain management facility OR pain management facilities; (5) pain center OR pain centre; (6) modality oriented clinic; (7) multidisciplinary pain center; (8) pill mill; (9) pain relief center; (10) pain management program; (11) pain management center; (12) pain service; (13) multidisciplinary pain clinic; (14) pain management unit; and (15) MPTF (common abbreviation for multidisciplinary pain treatment facility). These terms were identified through a preliminary review of research articles about pain clinics, including a widely-cited description of pain clinic typologies by Loeser et al. [16]. MAXQDA located 3591 keyword matches among the 263 articles (see Fig. 2), which we read for context to determine eligibility. Records were considered eligible for inclusion if they stated or proposed a definition of a pain clinic **OR** described unique procedures, standards, treatments or staff composition of pain clinics. Using these criteria, 209 full-text records were excluded. Of the remaining 54 full-text records, we excluded articles that replicated legal definitions of pain clinics. Our final sample consisted of 32 full-text records that defined pain clinics.

##### Grey literature

Our basic inclusion criteria for grey literature identified four professional organizations specializing in pain management: American Chronic Pain Association, American Pain Society, American Academy of Pain Medicine, and American Society of Anesthesiology. Using the search function within their websites, we scanned various sections of the website for documents or statements related to pain clinics. Given the purpose of this analysis, we were particularly interested in documents located on pages dedicated to “policy” or “advocacy” goals of the organizations. We applied the same inclusion/exclusion criteria from the *academic literature* search to identify definitions of pain clinics. This search yielded 3 records, which we reviewed, extracted, and merged with the definitions from the academic literature.

Final “definitions” from the academic and grey literature included descriptions or phrases that identified pain clinics as distinct health service entities. Additionally, we included general (i.e., nonspecific) mission statements, medical approaches, or goals of pain management facilities as “definitions” of pain clinics. Given that we sought broad conceptual definitions, we excluded descriptions of singular pain clinics (e.g. from a case study or proof-of-concept paper). In total, we obtained 35 records of pain clinic definitions: 32 from academic literature and 3 from the grey literature. Finally, we utilized an iterative process to review the definitions of pain clinics and code overarching themes (see Table 2).

**Table 2:** Pain clinic definition typologies

General Category	Sub-Categories	Definition	Sources
Provider-based	Supervision-based	Who owns, manages or oversees the clinic	[16, 47]
	Composition-based	Combination of staff	[16, 47, 49, 62–64]
Outcome-based (Aim-based)		Outcome/goal distinct from chronic pain management for general population	[16, 47, 49, 61–63, 65–68]
Treatment-based	Single-modality pain clinic	Single approach to pain treatment	[16, 46, 49, 68, 69]
	Multidisciplinary	Multiple approaches to pain treatment	[16, 46, 47, 55, 67, 69–71]
	Interdisciplinary	Integrate multiple approaches to pain treatment through coordinated team	[53, 55, 68, 72]
Marketing-based		Advertise themselves as location where pain is managed	[58, 64, 73, 74]
Patient Case Mix		Ratio of pain management patients to providers	[47]

#### Medical literature analysis

Two researchers (JR and RS) conducted an inductive analysis of the medical literature [19, 20] to develop a taxonomy of pain clinic definitions. While the initial search encompassed both peer-reviewed and grey literature, the analysis focused on the peer-reviewed literature (as the grey literature primarily re-iterated and referenced concepts already captured in peer-reviewed literature). The researchers followed an inductive approach to textual data analysis [20, 21], whereby the initial coding scheme was first developed from the content of a subset of the articles. Using this list, they proceeded to code all the articles, adding new codes when content arose that did not fit the initial list. They then consolidated codes into themes. These themes formed a taxonomy of pain clinic definitions (see Table 2). As a quality check, a third researcher (BA) then reviewed the resulting clinic definition taxonomy and discussed areas of inconsistency and disagreement with the rest of the research team.

### Identification of state policies

In the next phase, we identified pain clinic definitions in state law, coded these definitions using the taxonomy of pain clinic definitions from the medical literature, and then assessed relative frequency of codes.

#### State policy data collection

##### Law search

In order to identify legal definitions of pain clinics in U.S. state laws, we conducted a search of state statutes and regulations using Westlaw. This search was conducted in August 2017 and we included laws in effect in August 2017. We used the following search terms: “pain clinic”, “pain management clinic”, “pain management facility” and “pain management medical practice” in a search of all states. Our initial search obtained 237 state statute results from 28 states and 150 state regulation results from 18 states. We read through each law (statute or regulation) in the initial search and excluded those laws that met the following exclusion criteria: 1) laws that were irrelevant to regulating pain clinics, 2) laws not yet enacted (as indicated by Westlaw), 3) laws that had been repealed, declared unconstitutional or preempted by Federal law (as indicated by Westlaw), and 4) mere cross-references or indexes using the search terms lacking substantive text. We considered laws to be irrelevant to regulating pain clinics if they did not directly regulate pain clinics, even if they contained a definition of pain clinics. For example, if a law regulated pain management practitioners rather than pain clinics, then we excluded the law, even if a pain clinic definition was embedded within the law (e.g. if the law defined “pain management practitioner” as one who works within a pain clinic).^3^ Cross-references related laws that are not part of the law itself. After applying these exclusion criteria, 168 results remained: 77 statutes from 10 states, and 91 regulations from 10 states. We then excluded any result that did not meet at least one of the following criteria: 1) define pain clinics, or 2) describe safe harbor laws, meaning laws that either exempt certain pain clinics from regulations or exempt certain entities from the definition of “pain clinic.”^4^ After applying these exclusion criteria, the following results remained (*n* = 10 states): Florida [22, 23], Georgia [24–26], Kentucky [27], Louisiana [28–30], Mississippi [31], Ohio [32, 33], Tennessee [34–36], Texas [37–39], Wisconsin [40, 41], West Virginia [42–44].

#### State policy analysis

One researcher (BA) used the medical literature taxonomy to conduct a deductive content analysis of state pain clinic laws [20, 21, 45], specifically a) definitions of pain clinics found in state laws and b) state safe harbor laws (a combination of actual safe harbor laws, which define pain clinics not subject to regulation, and exceptions to pain clinic definitions). When appropriate, state pain clinic definitions and safe harbor laws were coded according to the taxonomy with more than one code. After coding state laws, the relative frequency of specific codes was then assessed to determine which medical literature definitions of pain clinics appeared most and least frequently in state law definitions and safe harbor laws, potentially indicating state policy makers’ priorities with respect to defining and regulating pain clinics. While mapping the medical literature definitions onto state laws, those medical literature codes that were not a perfect match and their variations were noted. Any areas where deductive application of codes was unclear were discussed with members of the research team (BBP, DW, RS), and the final results of the coding were reviewed by DW and RS.

## Results

**Fig. 3:**
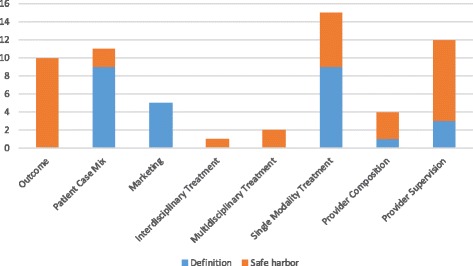
Frequency of Medical Definitions in State Law

Based on our review of the medical literature, we identified 5 categories of pain clinic definitions with 5 sub-definitions (see Table 2). The following medical literature definitions appear most frequently (see Fig. 3) within U.S. state law definitions of pain clinics: “patient case mix” (9/10 states); “single-modality treatment,” especially with respect to controlled substance use (9/10 states), and “marketing-based” (5/10). The “supervision-based” (3/10) and “composition-based” (1/10) categories appear infrequently. “Outcome-based,” “interdisciplinary treatment,” and “multidisciplinary treatment” definitional categories do not appear at all in state law definitions of pain clinics (but they do appear in state safe harbor laws). The following medical literature definitions appear most frequently within state safe harbor laws: “outcome-based” (10/10); “supervision-based” (9/10); and “single-modality treatment,” especially with respect to surgical or interventional methods (6/10). “Multidisciplinary treatment” (2/10), “composition-based” (3/10), “patient case mix” (2/10), and “interdisciplinary treatment” (1/10) definitions appear infrequently. No state safe harbor law included a “marketing-based” definition. Nine of ten states had pain clinic definitions that included at least two medical literature definitions, with an average of three categories. Only Texas’s pain clinic definition had a single medical definition category: “single-modality treatment.” In contrast, Wisconsin’s pain clinic definition included five definitional categories from the medical literature: “patient case mix,” “marketing,” “single-modality treatment,” “composition-based,” and “supervision-based.” Nine of ten state safe harbor laws had at least two medical literature definitions, with an average of three categories. The application of these definitions to existing state laws’ pain clinic definitions and safe harbor provisions are represented in Table 3. What follows is a description and examples of each of the pain clinic definitions from the medical literature and examples of laws to which the medical literature definitions were applied.

**Table 3:** Medical definitions applied to state pain clinic definition & “Safe Harbor” Laws

	LA	WV	WI	TX	TN	OH	GA	FL	MI	KY
Outcome	S	S	S	S	S	S	S	S	S	S
Patient Case Mix	D	D	D		D, S	D	D	D	D, S	D
Marketing			D		D		D	D		D
Treatment										
Interdisciplinary						S				
Multidisciplinary				S				S		
Single-modality	D	D, S	D	D	S	D, S	D	D, S	D, S	D, S
Provider										
Composition			D	S	S			S		
supervision	S	D, S	D	S	D, S	S	S	S	S	S

D: Pain clinic definitions in state law; S: Safe harbor provisions in state law

### Provider-based definitions

Provider-based definitions of pain clinics describe either the owners or supervisors of pain clinics, or a composition of providers within the pain clinic. For instance, Bonica [46] writes that one of the most efficient approaches to treatment is through “the team approach, or a pain clinic group composed of health professionals of different disciplines working and collaborating in a well-coordinated manner” (p.784). Bonica then describes an ideal composition of team members, including physicians, psychologists, and nurses, supervised by an experienced clinic coordinator. Therefore, we identified two subcategories of provider-based definitions: (a) supervision-based and (b) composition-based.

#### Supervision-based provider definitions

Supervision-based definitions of pain clinics characterize clinics based on who owns, manages or oversees them. The medical literature definition of “supervision” focuses on providers as owners or supervisors. For instance, de Meij [47] says, “a pain clinic or pain center should be run by an anesthesiologist specializing in pain management” (p.60). State definitions include institutions as owners or supervisors, such as hospitals, ambulatory centers, government agencies, and certain non-profit organizations or privately-owned clinics. All three states having supervision-based clinic definitions distinguished privately-owned clinics from publicly-owned clinics for purposes of regulation. For example, West Virginia’s definition states: “‘Pain management clinic’ means all privately-owned pain management clinics, facilities or offices not otherwise exempted from this article.” Nine states had a supervision-based safe harbor law, such as hospital ownership or state ownership. For example, Louisiana’s safe harbor law says: “The provisions of this Part shall not apply to any of the following: (5) A clinic maintained or operated by the United States or by any of its departments, offices, or agencies [48].”

#### Composition-based provider definitions

Composition-based definitions of pain clinics describe the combination of clinic staff, sometimes specifying training requirements or physician specialty. For example Castel et al. [49] described pain clinics with respect to the number and variety of health care providers working within the clinic. One state had a composition-based clinic definition and three states had a composition-based safe harbor law. For example, Florida’s safe harbor law describes staff qualifications needed for exemption from state regulation: “Each pain management clinic must register with the department unless…The clinic is wholly owned and operated by a physician multispecialty practice *where one or more board-eligible or board-certified medical specialists…perform interventional pain procedures of the type routinely billed using surgical codes* [emphasis added]*.*” [50].

### Outcome-based definitions

Outcome-based definitions capture clinics with outcomes or goals distinct from or in addition to the clinical treatment of chronic pain. Such outcomes may include education, research, acute pain management, palliative care for terminal diseases, or addiction treatment. For example, according to de Meij et al. [47], educational or training-based quality indicators of pain clinics include the following: “demonstrable active participation in scientific research by training: residents, fellows, healthcare providers involved in pain management, paramedical institutions, researcher with pain specialty”; their own “research program and research facilities (researcher, data registration)”; or “[f]ormal cooperation and relationship with an University Medical Center” (p. 61). Outcome-based state safe harbor laws distinguish between standalone outpatient clinics treating chronic pain and clinics associated with medical or dental schools, hospitals, hospice programs, clinical trial programs, nursing homes, or Suboxone clinics. These clinics have goals distinct from or in addition to the clinical management of chronic pain for the general population, such as education, acute pain management, terminal pain management, research, palliative care for the disabled or elderly, or addiction treatment, respectively. For example, Tennessee’s safe harbor law says: “This part does not apply to a nursing home [51].” Similarly, Louisiana’s safe harbor law says: “The provisions of this Part shall not apply to any of the following: A medical or dental school or outpatient clinic associated with a medical or dental school [52].” No state had an “outcome-based” clinic definition but all states had an “outcome-based” safe harbor law.

### Treatment-based definitions

Treatment-based definitions describe pain clinics by focusing on the treatment modalities offered and distinguishing between the scope and breadth of the treatment model, including the variety of treatment methods available and the integration of multiple treatment methods in patient care. For example, Bonica [46] states “although the terms ‘nerve block clinic’ and ‘pain clinic’ are often used interchangeably, there are significant differences between the two types of facilities. A nerve block clinic is a facility in which nerve blocks are done for the management of pain and other disease states, whereas a pain clinic has a much broader and more comprehensive scope” (p.785). Treatment-based definitions differ from composition-based definitions by focusing on the clinical treatment provided as opposed to the type of treatment provider. We identified three subcategories of treatment-based definitions: a) single-modality treatment; b) multidisciplinary treatment; and c) interdisciplinary treatment.

#### Single-modality treatment definitions

The single-modality treatment definition describes a single pain treatment approach, rather than a multidisciplinary or integrated approach. For example, some clinics only provide nerve blocks or anesthesia [49]. Single-modality treatment clinics are the most common type of pain clinics [53]. While the medical literature describes single-modality treatment clinics as those that provide *only* one type of treatment (e.g. nerve blocks), state laws with a single-modality treatment definition *focus* on a single treatment modality even though it is possible that other treatment methods are provided within the clinic as well. For example, Georgia’s law says, “Pain management clinic’ means…a medical practice or clinic with greater than 50% of its annual patient population being treated for chronic pain for nonterminal conditions by the use of Schedule II or III controlled substances [54].” Some clinics captured by this law provide treatments other than controlled substances (e.g. physical therapy), but Georgia has chosen to focus on the provision of controlled substances. Single-modality treatment safe harbor laws include laws exempting surgical facilities from regulations. Nine states had a single-modality clinic definition and six states had a single-modality safe harbor law. The single-modality safe harbor laws referenced non-opioid treatments, such as spinal nerve blocks or surgery. Every state with a single-modality clinic definition focused on narcotics as the single modality.

#### Multidisciplinary treatment definitions

The multidisciplinary treatment definition describes clinics using multiple approaches to pain treatment (e.g., physical and occupational therapy, behavioral strategies, and pharmacologic solutions). As compared to the interdisciplinary treatment definition, multidisciplinary treatment captures clinics provinging less coordinated care, with treatment occurring in “a parallel rather than as an integrated approach” [55] (p.2). No state had this definition, but two states had a multidisciplinary treatment safe harbor law. For example, Texas’s safe harbor law says: “This chapter does not apply to (7) a clinic owned or operated by a physician who treats patients within the physician’s area of specialty and *who personally uses other forms of treatment, including surgery, with the issuance of a prescription for a majority of the patients* [emphasis added]*”* [56].

#### Interdisciplinary treatment definitions

The interdisciplinary treatment definition describes clinics integrating multiple approaches to pain treatment with care coordinated between members of an interdisciplinary pain management team. Unlike multidisciplinary clinics, in which providers may work in different locations, team members of interdisciplinary clinics typically work under one roof, enabling communication and coordination [53]. No state had an interdisciplinary treatment clinic definition, and only Ohio had an interdisciplinary safe harbor law. Ohio’s law says: “‘Pain management clinic’ does not include any of the following: (vii) An interdisciplinary pain rehabilitation program with three-year accreditation from the commission on accreditation of rehabilitation facilities [57].”

### Marketing-based definitions

The marketing-based definition captures pain clinics that advertise as a location where pain is treated or managed. For example, Peng et al. [58] identify pain clinics partly based on how they advertise themselves. State laws that defined pain clinics with respect to advertising activity had a marketing-based definition. For example, Florida’s law says, “Pain-management clinic” or “clinic” means any publicly or privately owned facility (I) that advertises in any medium for any type of “pain-management services” [59]. Five states had a marketing-based clinic definition, and no state had a marketing-based safe harbor law.

### Patient case mix definitions

The patient case mix definition identifies facilities as pain clinics using a pre-established ratio or percentage of the number of patients being treated for pain per treatment provider. We have interpreted this definition to include the ratio of patients being treated for pain within the clinic relative to patients not treated for pain. State laws defining pain clinics as entities in which the majority of patients are treated for pain had a patient case mix definition. Nine states had a patient case mix clinic definition, and two states had a patient case mix safe harbor law. For example, Tennessee’s law says: “‘Pain management clinic’ or ‘clinic’ means a privately-owned clinic, facility or office in which any health care provider licensed under this title *provides chronic nonmalignant pain treatment to a majority of its patients* [emphasis added] for ninety (90) days or more in a twelve-month period [60].”

## Discussion

In U.S. state laws, pain clinic definitions appear primarily designed to identify clinics with potentially suspicious behavior. As a result, the most common types of pain clinic definitions are single-modality treatment definitions, which reference the of narcotics within the clinic, and marketing-based definitions. Those clinics captured by state law definitions must register with the state and become subject to extra oversight, including periodic inspections, presumably allowing the state to close clinics thought to be contributing more harm than good. In contrast, state law safe harbor provisions appear designed to identify clinics unlikely to have suspicious behavior due to quality indicators. Therefore, clinic definitions associated with quality indicators, such as outcome-based definitions (e.g. association with a medical school) and supervision-based definitions (e.g. hospital or government supervision) appear most frequently in state safe harbor law provisions. In contrast, no state has a marketing-based definition in its safe harbor law, probably because marketing is not a quality indicator. Additionally, the vast majority of states exempt clinics with outcome-based definitions, meaning those clinics with goals distinct from or in addition to chronic pain management, such as hospices and nursing homes. Presumably these clinics are unlikely to exhibit stereotypically suspicious behavior related to “pill mills.”

An inconsistency exists with respect to how state laws define pain clinics and how peer-reviewed medical articles define them. Medical articles are less concerned with suspicious versus non-suspicious behavior within pain clinics. Instead, medical articles define pain clinics primarily with respect to the combination of treatment methods, specifically whether the clinic provides interdisciplinary treatment, multidisciplinary treatment or single-modality treatment. Interdisciplinary treatment and multidisciplinary treatment are discussed more positively than single-modality treatment [16]. In contrast, only single-modality treatment definitions appear in state pain clinic definitions, and even within state safe harbor provisions, interdisciplinary and multidisciplinary definitions appear infrequently. The relative frequency of these definitions in state law suggests states are less concerned about the mixture of treatments provided (e.g. whether interdisciplinary treatment or single-modality) than treatment through narcotics. Therefore, even a hypothetical clinic within which most patients receive narcotic treatment in an interdisciplinary context (e.g. integrated with physical therapy, mental health counseling, and massage) is captured as being potentially suspicious simply due to the provision of narcotic treatment.

Interestingly, quality indicators associated with definitions in safe harbor laws are not required for clinics to operate, but rather exempt the clinic from regulation. For example, pain clinics are not required to provide multidisciplinary treatment. However, if clinics in two of the states included in this review provide multidisciplinary treatment, then the clinic becomes exempted from pain clinic regulations through the safe harbor law. Possibly, these exemptions incentivize clinics to provide multidisciplinary or interdisciplinary treatment, a consideration that future empirical research should explore. One could imagine a different style of regulation, such as that suggested by de Meij [47] for pain clinics in the Netherlands, in which only those pain clinics that meet certain quality indicators are permitted to operate. We do not comment on whether or not that would be an appropriate approach to pain clinic regulation in the United States, but rather note that no state laws operate in this manner.

Finally, nine of ten states incorporate multiple definitions in their pain clinic laws, and all states incorporate multiple definitions in their safe harbor laws, suggesting states have more than one conceptualization of pain clinics. Multiple legal definitions allow states to subject a larger percentage of clinics to state regulation.

Given inconsistencies between state laws and the medical literature identified, it appears state policy is not reflecting scientific consensus in focus or content. Such inconsistencies are not uncommon in public health, given that researchers make decisions based on specialized knowledge, while policymakers make decisions based on stakeholder interests and preexisting policies. Policymakers’ decisions often reflect short-term interests keyed to an election cycle [10]. With respect to pain clinic laws, stakeholders may currently demand increased narcotic prescribing oversight in light of the opioid overdose crisis rather than incentives for interdisciplinary pain management. Existing state laws’ emphasis on narcotic treatment (via regulation of single-modality treatment) and relative lack of emphasis on multidisciplinary or interdisciplinary treatment could perversely incentivize health care providers to avoid narcotic treatment even when medically advisable, while ignoring opportunities to provide multidisciplinary or interdisciplinary treatment.

Future work should discern the impact of legal pain clinic definitions and safe harbor exemptions on provider decision-making. For example, it is unknown whether providers actively try to avoid regulation by including features within their clinic identified by state safe harbor laws. It is also unclear whether providers are aware of state pain clinic regulations and exemptions to begin with. Additionally, research should discern the impact of state pain clinic definitions and safe harbor laws on the supply of opioids and opioid-related morbidity and mortality.

Regarding limitations, by focusing on academic literature rather than gray literature, we may have deemphasized interpretations of pain clinic definitions by governmental and professional organizations. Additionally, coding was a subjective qualitative process that might be subject to unconscious biases; however, our coding protocols likely reduced the potential for strong bias in the results. Finally, our research approach did not seek to identify whether medical literature definitions of pain clinics were utilized in the process of making state law; rather, our results merely suggest whether such definitions are reflected in the final law.

## Conclusion

This is the first study to our knowledge to compare pain clinic definitions found in the medical literature with those found in U.S. state laws. The inconsistencies we found demonstrate a lack of translation of scientific and professional knowledge into policy. Pain clinic definitions emphasized in the academic medical literature, especially those related to multidisciplinary and interdisciplinary treatment, did not feature prominently in state laws. States’ almost singular focus on narcotic prescribing in legal definitions ignores an opportunity for encouraging multidisciplinary or interdisciplinary treatment, such as by including multidisciplinary or interdisciplinary treatment in safe harbor exemptions. Future work should discern the impact of different legal pain clinic definitions on provider decision-making and state-level health outcomes.

## Footnotes

1 The literature on pain clinic definitions very much stems from these papers which were published in 1990 and 1991. Early searches which did not include these time parameters yielded few useful results, although a paper published before this date (Bonica 1977) was added to our final analysis based on expert input. Also, no state pain clinic regulations preceded the date of these seminal papers.

2 PDFs that were not readily available at our institution were submitted to our interlibrary loan service. Several of these came back as irretrievable from library staff while several others were simply not retrieved without notification from staff. Often these articles are irretrievable if they cannot be located in English, although the source is originally identified as English. Additionally, given that our search included “grey” literature, we expected some irregularities in the search and retrieval process of that literature.

3 We excluded state regulations of individual providers who treat chronic pain from review of pain clinic laws, as the two types of laws differ in scope and content. Additionally, some states regulate individual prescribers but not pain clinics. Regulations of individual providers focus on prescribers of opioids for chronic pain. These regulations capture a very broad swath of providers, as they are applicable any time a provider prescribes opioids for chronic pain, regardless provider specialty. On the other hand, pain clinic regulations are much narrower, typically only applying to entities that market themselves as treating pain, that frequently prescribe controlled substances for pain, or that have a majority of patients treated for pain. Second, the content of state regulations of individual providers differs from that of pain clinic regulations. Pain clinic regulations require separate registration for pain clinics without which the pain clinic cannot legally operate, improving state oversight of such clinics and subjecting clinics to state inspection. Regulations detail management and structure of the business, rather than or in addition to treatment practices for pain management. In contrast, regulations of individual providers do not require practitioners to register with the state, nor do they subject practitioners to inspection. They focus almost exclusively on how to provide pain management treatment with narcotics, detailing maximum opioid dosage, number of days per prescription, required consultations with specialists, and/or required physical examinations. Such laws are far more common than laws specifically regulating pain clinics.

4 As used in this paper, the term “safe harbor law” has two meanings. First, it refers to any state law provision that explicitly exempts certain pain clinics from regulations, even though these clinics fall within the definition of “pain clinic.” For example, a state law exempting pain clinics associated with hospitals from regulations is a safe harbor law. Second, “safe harbor law” also refers to state law provisions that explicitly exclude certain types of clinics from the definition of “pain clinic.” For example, a state law explicitly exempting addiction treatment clinics from the definition of pain clinics is a safe harbor law.

## References

[1] Nahin RL (2015). Estimates of pain prevalence and severity in adults: United States, 2012. J Pain.

[2] Muhuri P, Gfroerer J, Davies M. Associations of nonmedical pain reliever use and initiation of heroin use in the United States: CBHSQ Quarterly Review. Rockville: 2013.

[3] Compton W, Jones C, Baldwin G (2016). Relationship between nonmedical prescription-opioid use and heroin use. NEJM.

[4] Centers for Disease Control and Prevention [CDC] (2016). Wide-ranging online data for epidemiologic research (WONDER).

[5] Centers for Disease Control & Prevention. Opioid Data Analysis: Atlanta CDC, National Center for Health Statistics. 2016. https://www.cdc.gov/drugoverdose/data/analysis.html. Accessed 1 Dec 2017.

[6] Centers for Disease Control and Prevention [CDC] . (2011). Policy Impact: Prescription Painkiller Opioid Overdoses. Atlanta, GA: CDC, National Center for Injury Prevention and Control. Available at https://www.cdc.gov/drugoverdose/pdf/policyimpact-prescriptionpainkillerod-a.pdf.

[7] Wickramatilake S, Zur J, Mulvaney-Day N, von Klimo MC, Selmi E, Harwood H (2017). How states are tackling the opioid crisis. Public Health Rep.

[8] Prescription Drug Monitoring Program Training & Technology assistance center. State profiles. 2017. http://www.pdmpassist.org/content/state-profiles. Accessed 1 Dec 2017.

[9] Dowell D, Zhang K, Noonan RK, Hockenberry JM (2016). Mandatory provider review and pain clinic laws reduce the amounts of opioids prescribed and overdose death rates. Health Aff.

[10] Brownson RC, Royer C, Ewing R, McBride TD (2006). Researchers and policymakers: Travelers in parallel universes. Am J Prev Med.

[11] Brownson RC, Gurney JG, Land GH (1999). Evidence-based decision making in public health. J Public Health Manage Pract.

[12] MAXQDA. Software for qualitative data analysis, 1989–2017. Berlin: VERBI Software – Consult- Sozialforschung GmbH.

[13] Gough D, Thomas J, Oliver S. Clarifying differences between review designs and methods. Syst Rev. 2012;1(1) 10.1186/2046-4053-1-28. 10.1186/2046-4053-1-28 PMC3533815 22681772

[14] Russell CL. An overview of the integrative research review. Prog Transplant. 2005;15(1):8-13. 10.1177/152692480501500102 15839365

[15] Torraco RJ (2005). Writing integrative literature reviews: guidelines and examples. Hum Resour Dev Rev.

[16] Loeser J. Desirable characteristics for pain treatment facilities: report of the IASP taskforce. In: Bond MR, Woolf CJ, editors. Proceedings of the VIth world congress on pain: Elsevier science publishers BV; 1991. p. 411–5.

[17] JCD WELLS, JB MILES (1991). Pain clinics and pain clinic treatments. Br Med Bull.

[18] EndNote, reference management software. Clarivate analytics. Philadelphia; 2017.

[19] Watson DP, Shuman V, Kowalsky J, Golembiewski E, Brown M (2017). Housing first and harm reduction: a rapid review and document analysis of the US and Canadian open-access literature. Harm Reduct J.

[20] Bowen GA (2009). Document analysis as a qualitative research method. Qualtative Res J.

[21] Kuckartz U (2014). Qualitative text analysis: a guide to methods, practice, and using software.

[22] FLA. STAT ANN. § 458.3265 (eff. 2016).

[23] FLA. STAT ANN. § 459.0137 (eff. 2016).

[24] GA Code ANN. . § 43–34-282 (eff. 2013).

[25] GA. COMP. R. & REGS. 360–8-.01.

[26] GA. COMP. R. & REGS. 360–8-.07.

[27] KY. REV. STAT ANN. § 218A.175 (eff. 2015).

[28] LA. REV. STAT. ANN. § 40:2198.12 (eff. 2014).

[29] LA. REV. STAT. ANN. § 40:2198.11 (eff. 2006).

[30] LA. ADMIN. CODE tit. 48, pt. I, § 7801.

[31] 30-17 MISS. Code r. § 2640:1.2(G) (eff. 2012).

[32] OHIO REV. Code ANN. § 4731.054 (eff. 2013).

[33] OHIO ADMIN. CODE 4731–29-01 (eff. 2017).

[34] TENN. Code ANN. § 63–1-301 (eff. 2017).

[35] TENN. Code ANN. § 63–1-302 (eff. 2017).

[36] TENN. COMP. R. & REGS. 1200–34-01-.05.

[37] TEX OCC. Code ANN. § 168.001 (eff. 2015).

[38] TEX OCC. Code ANN. § 168.002 (eff. 2014).

[39] 22 TEX. ADMIN. CODE §195.1 (eff. 2016).

[40] WIS. STAT. ANN. § 50.60 (eff. 2016).

[41] WIS. STAT. ANN. § 50.65 (eff. 2016).

[42] W. VA. Code ANN. § 16-5H-2 (eff. 2016).

[43] W. VA. Code ANN. § 16-5H-5 (eff. 2016).

[44] W. VA. CODE STAT. REG. §69–8-3.

[45] Hsieh H-F, Shannon SE (2005). Three approaches to qualitative content analysis. Qual Health Res.

[46] Bonica JJ (1977). Basic principles in managing chronic pain. Arch Surg.

[47] de Meij N, van Grotel M, Patijn J, van der Weijden T, van Kleef M. First Dutch Consensus of Pain Quality Indicators for Pain Treatment Facilities. Pain Pract. 2016;16(1):57–66. 10.1111/papr.12331 26200939

[48] LA. REV. STAT. ANN. § 40:2198.12(E)(5) (eff. 2014).

[49] Castel LD, Freburger JK, Holmes GM, Scheinman RP, Jackman AM, Carey TS (2009). Spine and Pain clinics serving North Carolina patients with back and neck pain: what do they do, and are they multidisciplinary?. Spine (Phila Pa 1976).

[50] FLA. STAT. ANN. § 459.0137(1)(a)(2)(h) (eff. 2016).

[51] TENN. Code ANN. § 63–1-302(4) (eff. 2017).

[52] LA. REV. STAT. ANN. § 40:2198.12(E)(1) (eff. 2014).

[53] Jay G (2016). Interdisciplinary Treatment of Chronic Noncancer Pain. Clinician’s Guide to Chronic Headache and Facial Pain.

[54] GA. Code ANN. § 43–34-282(7) (eff. 2013).

[55] Turk D, Palce J, Cowan P, Stanos S, Jamison R, Covington E. et al. Interdisciplinary Pain Management. http://americanpainsociety.org/uploads/about/position-statements/interdisciplinary-white-paper.pdf. Accessed 1 Dec 2017

[56] TEX OCC. Code ANN. § 168.002(8) (eff. 2014).

[57] OHIO ADMIN. CODE 4731–29-01(A)(7)(g) (eff. 2017).

[58] Peng P, Choiniere M, Dion D, Intrater H, Lefort S, Lynch M, Ong M, Rashiq S, Tkachuk GVY, Peng P, Choiniere M, Dion D, Intrater H, Lefort S (2007). Challenges in accessing multidisciplinary pain treatment facilities in Canada. Can J Anaesth.

[59] FLA. STAT. ANN. § 459.0137(1)(a)(1)(c) (eff. 2016).

[60] TENN. COMP. R. & REGS. 1200–34-01-.05(1).

[61] Wells JC, Miles JB. Pain clinics and pain clinic treatments. Br Med Bull. 1991;47(3):762–85. 10.1093/oxfordjournals.bmb.a072506 1794083

[62] Tyrer S, Lievesley A (2003). Pain following traumatic brain injury: assessment and management. Neuropsychol Rehabil.

[63] Fidahić M, Dogan K, Sapunar D, Puljak L (2015). National survey of pain clinics in Croatia: organization and services. Acta Med Acad.

[64] Peng P, Stinson J, Choiniere M, Dion D, Intrater H, LeFort S, et al. Dedicated multidisciplinary pain management centres for children in Canada: the current status. Can J Anaesth. 2007;54(12):985-91. 10.1007/BF03016632 18056207

[65] Campbell C, Guy A. “Why can”t they do anything for a simple back problem?’ A qualitative examination of expectations for low back pain treatment and outcome. J Health Psychol. 12:641–52. 10.1177/1359105307078171. 10.1177/1359105307078171 17584815

[66] Okifuji A, Turk DC (1998). Philosophy and efficacy of multidisciplinary approach to chronic pain management. J Anesth.

[67] Twillman R.: Commentary Pill Mills Are Not Pain Clinics— The Challenge of Addressing One Without Harming the Other. J Med Regul. 2012;98:7–11. http://jmr.fsmb.org/Archive/2010s/Social Media and Social Networking in Medical Practice.pdf. Accessed 1 Dec 2017.

[68] Commission on Accreditation of Rehabilitation Facilities (2017). Medical Rehabilitation Program Descriptions..

[69] Vasudevan SV, Lynch NT. Pain centers-organization and outcome. Rehabil Med. 1991;154:532–5. PMC1002821 1866945

[70] Harding G, Campbell J, Parsons S, Rahman A, Underwood M (2010). British pain clinic practitioners’ recognition and use of the bio-psychosocial pain management model for patients when physical interventions are ineffective or inappropriate: results of a qualitative study. BMC Musculoskelet Disord.

[71] Fisbain DA, Rosomoff HL, Steele-Rosomoff R, Cutler BR (1995). Types of pain treatment facilities and referral selection criteria. A review. Arch Fam Med.

[72] Okifuji A (2003). Interdisciplinary pain management with pain patients: evidence for its effectiveness. Semin Pain Med.

[73] Morley-Forster PK (2007). Tomorrow and tomorrow and tomorrow:wait times for multidisciplinary pain clinics in Canada. Can J Anesth.

[74] Peng P, Stinson J, Choiniere M, Dion D, Intrater H, Lefort S, et al. Role of health care professionals in multidisciplinary pain treatment facilities in Canada. Pain Res Manag. 2008;13(6):484–8 10.1155/2008/726804 PMC2799317 19225605

